# Pathological Functions of LRRK2 in Parkinson’s Disease

**DOI:** 10.3390/cells9122565

**Published:** 2020-11-30

**Authors:** Ga Ram Jeong, Byoung Dae Lee

**Affiliations:** 1 Department of Neuroscience, Kyung Hee University, Seoul 02447, Korea; garamgogo@naver.com; 2 Department of Physiology, Kyung Hee University School of Medicine, Seoul 02447, Korea

**Keywords:** Parkinson’s disease, LRRK2, α-synuclein, neurodegeneration, Lewy body

## Abstract

Mutations in the gene encoding leucine-rich repeat kinase 2 (LRRK2) are common genetic risk factors for both familial and sporadic Parkinson’s disease (PD). Pathogenic mutations in LRRK2 have been shown to induce changes in its activity, and abnormal increase in LRRK2 kinase activity is thought to contribute to PD pathology. The precise molecular mechanisms underlying LRRK2-associated PD pathology are far from clear, however the identification of LRRK2 substrates and the elucidation of cellular pathways involved suggest a role of LRRK2 in microtubule dynamics, vesicular trafficking, and synaptic transmission. Moreover, LRRK2 is associated with pathologies of α-synuclein, a major component of Lewy bodies (LBs). Evidence from various cellular and animal models supports a role of LRRK2 in the regulation of aggregation and propagation of α-synuclein. Here, we summarize our current understanding of how pathogenic mutations dysregulate LRRK2 and discuss the possible mechanisms leading to neurodegeneration.

## 1. Introduction

Parkinson’s disease (PD) is the second most common neurodegenerative disease among the elderly population. The cardinal clinical symptoms of PD include resting tremors, muscular rigidity, bradykinesia, postural instability, and gait problems. Patients with PD often present unilateral motor symptoms that eventually become bilateral as the disease progresses [1]. Because motor symptoms predominantly result from the prominent loss of dopaminergic (DA) neurons in the substantia nigra pars compacta (SNpc), current treatment strategies focus on dopamine replacement. Although the diagnosis of PD relies on clinical features derived from dopamine deficiency, it has long been recognized that pathology underlying PD involves several brain areas beyond the dopaminergic system, and that pathology also extends into the peripheral autonomous nervous system. Indeed, the majority of PD patients show a variety of non-motor symptoms, including hyposmia, sleep disruption, and constipation, and non-motor dysfunctions commonly precede motor symptoms by several years [2]. This has motivated clinical studies to assess olfactory dysfunction or rapid eye movement sleep behavior disorder as potential risk factors of developing PD [3]. Other non-motor symptoms, such as dementia, cognitive dysfunction, and hallucinations arise later in the disease. Collectively, the complicated clinical features imply that a broad spectrum of neurophysiological mechanisms are involved in PD pathology. 

The majority of PD cases are idiopathic, while 5–10% of PD cases are familial and linked to mutations in multiple genes, such as α-synuclein (*SNCA*), Parkin (*PRKN*), PTEN-induced putative kinase 1 (*PINK1*), *DJ-1*, *VPS35*, glucocerebrosidase (*GBA*), and leucine-rich repeat kinase 2 (LRRK2) [4]. Among the PD genes, LRRK2 mutations comprise the most frequent cause of familial PD cases and are major risk factors for idiopathic PD cases. The most common mutation LRRK2-G2019S accounts for up to 6–40% of familial cases, depending on the ethnic group, and up to 2% of all sporadic cases [5,6,7,8]. Moreover, LRRK2-associated PD is generally indistinguishable from sporadic PD in terms of the age of onset, disease progression, and motor symptoms. Therefore, deciphering the role of LRRK2 might provide important insights into understanding molecular mechanisms of both familial and sporadic PD etiology and developing disease-modifying treatments for PD. 

PD pathology is generally characterized by the preferential loss of DA neurons in SNpc and the presence of proteinaceous inclusions known as Lewy bodies (LBs). Enormous efforts have been made to understand the mechanisms by which each feature contributes to the pathology and progression of PD. Increasing lines of evidence suggest that the cardinal neuropathological features are not separate events. Molecular components, pathways, or mechanisms involved in each feature interact, and their interactions contribute to the pathogenesis of PD. In this review, we briefly discuss evidence regarding the role of LRRK2 in the regulation of key neuropathological features, and discuss a possibility that LRRK2 plays a role as an important modulator that mediates the interactions. 

## 2. LRRK2 in Neurodegeneration

### 2.1. The Structure of LRRK2 and Regulation of Enzymatic Activities

LRRK2 is a large, multidomain protein (280 KDa) and belongs to the ROCO superfamily of proteins. It consists of multiple protein–protein interaction domains, such as N-terminal armadillo, ankyrin, and leucine-rich repeat domains, and a C-terminal WD40 domain [9]. An interesting feature of LRRK2 is that it contains two distinct enzymatic domains, a Ras of complex (ROC) GTPase domain and a serine/threonine kinase domain, separated by a C-terminal of ROC (COR) domain. Familial mutations tend to cluster within the catalytic domains of LRRK2. The kinase activity of LRRK2 has been assessed by measuring the autophosphorylation of LRRK2 [10], LRRK2 phosphorylation at Ser 1292, [11] and/or phosphorylation of an artificial peptide substrate—LRRKtide [12], or myelin basic protein (MBP) as a generic kinase substrate [13]. In addition to kinase activity, LRRK2 exhibits GTPase activity, presenting the capacity of GTP binding and hydrolysis in in vitro assay [14,15].

Molecular mechanisms that regulate the kinase and the GTPase activities of LRRK2 are not completely elucidated, but both inter- and intra-molecular regulation have been suggested to control enzymatic activities. LRRK2 forms a dimer in cells, and dimerization may be a critical step for the regulation of its enzymatic activities and membrane localization [16,17,18,19,20,21]. LRRK2 dimers show higher kinase activity compared to LRRK2 monomers, and the GTPase reaction also seems to depend on dimerization [22]. Furthermore, LRRK2 dimers are enriched in membrane fractions, and membrane LRRK2 shows enhanced kinase activity compared to cytosolic LRRK2 [20]. Biochemical analyses using truncated forms of LRRK2 and structural modeling studies with full length LRRK2 dimers suggest that the ROC and the COR domains function as primary dimerization interfaces [19]. It has been suggested that GTPase and kinase domains of LRRK2 could be regulated via an intramolecular mechanism. Missense mutations, such as K1347A and K1348N in the P-loop of the ROC domain of LRRK2, disrupt guanine nucleotide binding and impair LRRK2 autophosphorylation (although the caveat is that the mutations also inhibit dimerization) [23]. Conversely, LRRK2 kinase activity has been shown to regulate the GTPase activity through autophosphorylation of the ROC domain [24,25,26]. Kinase activity of LRRK2 can be regulated by guanine nucleotide exchange factor (GEF) or GTPase activating protein (GAP), such as ArfGAP1, RGS2, and ArhGEF7 [27,28,29,30]. 

### 2.2. Functions of Pathogenic LRRK2 Mutations in Neurodegeneration

Various genetic variants of LRRK2 have been identified and several missense mutations (N1437H, R1441C/G/H, Y1699C, G2019S and I2020T) are considered to be pathogenic. Those mutations segregate with disease in PD families, and pathophysiological roles have been suggested in a number of cellular and animal models [31]. Of note, pathogenic mutations are located in the catalytic domains: G2019S and I2020T mutations in the kinase domain; N1437H and R1441C/G/H in the ROC domain; and Y1699C in the COR domain. Thus, many studies that aim to understand the molecular and cellular mechanisms of pathogenic LRRK2 mutants have focused on elucidating whether and how the mutations change enzymatic activities, how they affect LRRK2 function, and how they contribute to the pathophysiology of PD. LRRK2 G2019S, the most common genetic determinant of PD, exhibits higher kinase activities compared to LRRK2 wild type (WT) [10,12,15,32,33,34]. The effect of I2020T mutation on LRRK2 kinase activity has been rather controversial, with some reporting increased activity [10,32], while others have shown decreased activity [12], and others have even described no changes in the kinase activity. ROC-COR domain mutations, LRRK2 R1441C/G/H and Y1699C show impaired GTP hydrolysis and enhanced GTP-binding [15,35] compared to LRRK2 WT. LRRK2 R1441C/G and Y1699C show increased kinase activity [15], whereas kinase domain mutants, LRRK2 G2019S and I2020T do not alter GTP binding [15,23]. 

The relationship between LRRK2 enzymatic activities and PD pathology has been and continues to be the subject of intensive investigation and has been tested in various PD model systems. In primary neuronal cultures, overexpression of LRRK2 G2019S, I2020T, R1441C, or Y1699C consistently induced neuronal toxicity, as evidenced by neurite shortening, cell death, or impaired functions of intracellular organelles [13,15,23,34,36,37,38,39]. Furthermore, many of these phenotypes were shown to be alleviated by introducing kinase-inactive or GTP binding-deficient mutations and/or treatment with chemical inhibitors of LRRK2 [13,40]. Several LRRK2 transgenic (Tg) animal models have been developed to recapitulate pathological phenotypes of PD. In *Drosophila* LRRK2 Tg models, Tg lines expressing LRRK2 G2019S, R1441C, Y1699C, or I2020T commonly exhibit age-dependent DA neuronal loss, disruption of dopamine homeostasis, locomotor defects or reduced survival [41,42,43,44,45]. *Caenorhabditis elegans* (*C. elegans*) LRRK2 Tg models expressing LRRK2 G2019S or R1441C also show neurodegeneration of DA neurons, reduced dopamine levels, and locomotor dysfunction [45,46]. These toxic gain-of-function effects of familial LRRK2 mutations are abolished by LRRK2 inhibitors. Liu et al. provided evidence that the administration of LRRK2 kinase inhibitors could rescue the toxic effects of LRRK2 G2019S in both *Drosophila* and *C. elegans* models [45]. In rodent models, herpes simplex viral- (HSV) or adenoviral-mediated expression of LRRK2 G2019S in the striatum of mice or rats induced robust degeneration of DA neurons in SNpc [13,47]. By contrast, the kinase-inactive variant LRRK2, G2019S/D1994A did not induce neurodegeneration, and neurodegeneration induced by HSV-LRRK2 G2019S was prevented by the administration of pharmacological inhibitors of LRRK2 kinase. Until now, several Tg rodent models overexpressing pathogenic LRRK2 mutants have been developed to mimic certain aspects of PD, such as selective DA neuronal loss, disruption of dopamine homeostasis, locomotor deficit or/and accumulation of tau and α-synuclein [48,49,50,51,52]. Knock-in (KI) models expressing a PD-associated LRRK2 mutation at endogenous levels did not show overt DA neuronal cell loss, but the mice at old age showed altered dopamine homeostasis, dysregulation of dopamine transport and accumulation into the synapse, and mild behavioral deficits, which are related to the prodromal phase of PD [53,54]. In fact, despite the fact that some models show impaired dopaminergic neurotransmission [55,56] and mild parkinsonian motor features at late stages [57], the majority of LRRK2 KI mice do not exhibit selective loss of DA neurons from the SNpc, the hallmark histopathological feature of PD [50,54,55,56,58,59]. In general, the pathological phenotypes in LRRK2 Tg or KI mice is highly dependent on aging and expression levels in DA neurons. Nonetheless, numerous cellular and animal LRRK2 models have repeatedly shown that LRRK2 exerts neuronal toxicity through kinase-dependent mechanisms. These observations attracted the interest of basic scientists and pharmacological companies to investigate how the kinase activity of LRRK2 in controlled. 

### 2.3. Kinase Substrates of LRRK2 and Their Roles in Neurodegeneration

A considerable amount of work has been undertaken to identify the physiological substrates of LRRK2 kinase. LRRK2 has been suggested to play multiple functions, ranging from cytoskeletal remodeling to protein expression, to synaptic transmission, and to membrane trafficking, through the phosphorylation of diverse substrates (Table 1) [60,61,62,63,64,65,66], such as moesin [12], β-tubulin [67], tau [68], microtubule affinity-regulating kinase 1 (MARK1) [69], Futsch [70], FoxO1 [71], 4E-BP1 [72], ribosomal protein s15 (RPS15) [73], ArfGAP1 [27], endophilin A1 [74], snapin [75], RGS2 [29], N-ethylmaleimide sensitive fusion (NSF) [76], synaptojanin-1 [77], and a subset of Rab GTPases [78,79,80]. Although most of these substrates have been shown to be directly phosphorylated by LRRK2 in in vitro kinase assays, it is not clear if they are phosphorylated in a cellular context or in vivo. Phosphorylation of endogenous Rab GTPases (Rab3, Rab8, Rab10, Rab12, Rab35, and Rab43) by LRRK2 in cells and the mammalian brain has been validated by several groups [79,80].

Early studies demonstrated that the expression of the pathogenic mutants of LRRK2 caused marked shortening of neurites, whereas depletion of LRRK2 increased neurite length and branching in primary cultures of embryonic cortical neurons [13,15,34,39]. Many LRRK2 substrates, such as moesin, β-tubulin, tau, and MARK1 have been suggested to play key roles in neurite extension via the regulation of cytoskeletal structures and dynamics. In cell-free assays, microtubule polymerization was enhanced by incubation with LRRK2 G2019S compared to LRRK2 WT or kinase-inactive LRRK2 D1994A [67]. It has been shown that tau, a microtubule-association protein (MAP), physically associates with and can be phosphorylated by LRRK2, in the presence of tubulin. In physiological conditions, tau associates with neuronal microtubules and is thought to play critical roles in the regulation of microtubule stability and dynamics [81,82,83]. In pathological conditions, tau detaches from microtubules and forms aggregates. Deposition of tau aggregates is a hallmark of Alzheimer’s disease, but is also found in a variety of other neurodegenerative diseases, including frontotemporal dementia with parkinsonism-17, Pick disease, progressive supranuclear palsy and corticobasal degeneration [84,85]. Phosphorylation induces tau to dissociate from microtubules, and hyperphosphorylation of tau has been proposed to contribute to neurodegeneration by the loss of microtubule stabilizing function of tau and by promoting tau aggregate formation. Increased phosphorylation of tau have been reported in multiple cellular and animal models expressing pathogenic LRRK2 mutants and in the postmortem brains from LRRK2-linked PD patients [39,86,87,88], although it is not clear whether the phosphorylation is directly mediated by LRRK2. It is plausible that hyperactivity of pathogenic LRRK2 plays a role in neurodegeneration by disturbing microtubule dynamics, but future studies are needed to investigate if LRRK2 phosphorylates tubulin and tau in vivo and whether and how the phosphorylation events contribute to PD pathology.

A group of LRRK2 substrates, such as endophilin A1, snapin, synaptojanin-1, and NSF, are key regulators of synaptic transmission. In neurons, synaptic transmission requires precisely controlled membrane trafficking at the presynaptic terminal in order to recover and recycle membranes that have fused with the plasma membrane during neurotransmitter release. Through postmortem brain studies of PD patients, synaptic dysfunction has been suggested to represent early events in PD [89]. Furthermore, multiple lines of evidences support the notion that LRRK2 plays a role as a regulator of clathrin-dependent endocytosis and recycling of synaptic vesicles [90]. In the course of synaptic vesicle endocytosis (SVE), endophilin promotes an early step by generating membrane curvature and a later step involved in vesicle release at the presynaptic membrane. Phosphorylation of endophilin by LRRK2 has been suggested to modulate the membrane deformation and release of endocytic vesicles [74]. In addition to SVE, LRRK2 can modulate synaptic vesicle fusion by regulating the dissociation of the SNARE complex via NSF phosphorylation [76]. NSF is an ATPase that catalyzes the release of SNARE complexes, thus allowing SVE and the next cycle of fusion. Phosphorylation of NSF by LRRK2 enhanced the catalytic activity of NSF and thus increased the rate of SNARE complex disassembly. These results imply that LRRK2 may tune the kinetics of synaptic vesicle recycling via NSF phosphorylation. Apart from endophilin and NSF, LRRK2 has been reported to bind and phosphorylate many other synaptic vesicle proteins [91], suggesting a broad role in synaptic transmission. 

To date, it has been reported that LRRK2 can phosphorylate fourteen Rab GTPases (Rab3a/b/c/d, Rab5a/b/c, Rab8a/b, Rab10, Rab12, Rab29, Rab35, and Rab43) at a conserved residue, which is located in the switch II effector-binding motif [78,79,80]. The Rab GTPase family consists of more than 60 members in the human genome, and functions as a molecular switch in the regulation of intracellular vesicle trafficking. Rab GTPases cycle between the inactive GDP-bound and the active GTP-bound forms at specific membranous compartments. The rate of GDP/GTP cycling is regulated by specific GAPs and GEFs, which activate and inactivate the GTPase activity. GEF promotes GTP binding by inducing the dissociation of GDP from Rab GTPases and causes major conformational changes in two highly flexible regions, switch I and II. The conformational change enables the binding of γ-phosphate and interaction with regulatory proteins and effectors [92]. In the case of Rab8a, phosphorylation by different pathogenic LRRK2 mutants decreases the affinity for guanine dissociation inhibitor (GDI), which is required for membrane delivery and recycling of Rab, and the phosphorylation eventually disrupts the balanced membrane-cytosol distribution of Rab8a [78]. Steger et al. replaced the LRRK2 target site in all fourteen LRRK2 substrate Rabs with either a phosphomimetic glutamic acid or a non-phosphorylatable alanine residue and examined how the mutation affected partner protein binding. Interestingly, non-phosphorylatable mutants of Rabs stably bound to GDI1/2 and Rab escort proteins, CHM and CHML, whereas the phosphomimetic mutations strongly prevented partner binding [79]. These results imply that Rab phosphorylation by LRRK2 modulates the binding with regulators of the GDP/GTP cycle or downstream effector molecules, which may affect subcellular localization and function. Some studies have suggested that phosphorylation of Rab8a and Rab10 by pathogenic mutants of LRRK2 caused defects in primary cilia formation [79,93] and centrosomal cohesion [94,95]. Both ciliogenesis and centrosomal cohesion were regulated by phosphorylation-dependent recruitment of their effector, RILPL1. Rab8a/Rab10 phosphorylation by LRRK2 has also been suggested to be involved in endolysosomal trafficking [96] and lysosomal homeostasis [97]. Upon lysosomal overload stress, LRRK2 was activated and recruited onto lysosomes, where LRRK2 stabilized Rab8 and Rab10 through phosphorylation, and Rab8/10 further recruited their effectors, EH domain-binding protein 1 (EHBP1) and EHBP1-like 1 (EHBP1L1), to regulate stress-induced lysosomal enlargement and secretion [97]. Jeong et al. provided important clues regarding the pathological consequences of Rab phosphorylation by LRRK2 by using neuronal culture and in vivo models [80]. In primary cultures of cortical neuron, non-phosphorylatable and phosphomimetic mutants of Rab1a, 3c, and 35, but not the WTs, specifically induced neuronal toxicity. Furthermore, intracranial injection of adeno-associated viral (AAV) vectors expressing non-phosphorylatable or phosphomimetic mutants of Rab35 into SNpc of mouse brains caused profound DA neuronal loss. Of note, increased Rab35 expression was detected in the SN region of multiple PD animal models, including LRRK2 G2019S and R1441C Tg mice and MPTP and rotenone intoxication mice, as well as in the serums of PD patients when compared to age-matched subjects [98]. Rab35 has been reported to be localized in the plasma membrane and the endosome. Rab35 seems to play a role in delivering internalized cargos to late endosomes or multivesicular bodies (MVBs) directed either for lysosomal degradation or for secretion through exosomes [99,100]. Endolysosomal system dysfunctions have been repeatedly reported in a diverse range of neurodegenerative diseases, and defects in the endolysosomal trafficking may represent early events during the progression of PD [101]. Notably, LRRK2 is enriched in membrane-associated fractions, including the Golgi apparatus, endoplasmic reticulum, mitochondria, multivesicular bodies (MVBs), lysosomal and endosomal vesicles, and autophagic vacuoles, suggesting a role of LRRK2 in membrane trafficking [102]. Studies from LRRK2 knock-out (KO) animals support a role in autophagy and lysosomal function [103,104,105]. Investigating the interaction between LRRK2 and Rab GTPases in various membranous organelles and how Rab phosphorylation by LRRK2 affects the endolysosomal and autolysosmal pathways will enhance our understanding of LRRK2-mediated neuropathologies.

## 3. Functions of LRRK2 in Lewy Pathology and Synucleinopathies

### 3.1. Lewy Pathology in PD

The presence of LBs, the proteinaceous inclusions characterized by the accumulation of misfolded and aggregated α-synuclein, is the main histopathological hallmark of PD. LBs are widely distributed in multiple brain regions, including the mesostriatal system, cortex, thalamus, hypothalamus, olfactory bulb and brain stem [106]. The morphology of LBs varies depending on the location in the brain (brainstem, limbic, or neocortical), and the heterogeneity may reflect the maturation stage and/or the biochemical variability of Lewy pathology [106,107]. LBs are also found in neurites, mainly in axons, referred to as intraneurite LBs or Lewy neurites. Misfolded and aggregated forms of α-synuclein are main protein components of LBs. It has been proposed that α-synuclein exists in a dynamic equilibrium of the unfolded monomers and helically folded tetramers, and that chronically shifting the physiological tetramers to excess monomers is associated with PD-like states [108]. Under pathological conditions, α-synuclein monomers become aggregated and initiate the formation of protofibrils and insoluble fibrils [109]. WT and disease-linked mutants of α-synuclein spontaneously form amyloid-like fibrils during prolonged incubation in vitro [110], but few inclusion bodies are found in various Tg mice overexpressing α-synuclein. Molecular mechanisms that trigger the fibrillization of α-synuclein and the formation of LBs in the brain remain poorly understood. Identification of the molecular components of LBs may provide important clues regarding the mechanisms of LB formation and disease progression. Proteomic analyses have identified more than 300 proteins in LBs, and about a hundred proteins have been validated by immunohistochemical analyses in various postmortem studies [106,111,112]. Notably, LRRK2 [36,113,114,115] has also been detected in LBs together with other PD-linked gene products, such as DJ-1 [116,117], Parkin [118], and PINK-1 [119]. In addition to proteinaceous components, LBs also contain non-proteinaceous (lipid) materials. Using correlative light and electron microscopy and tomography on postmortem human brain tissue from PD brain donors, one study [107] has revealed that α-synuclein-positive LBs and Lewy neurites contained a crowded mix-up of dysmorphic organellar and membranous features. Detection of LRRK2 and distorted membranous organelles in α-synuclein-positive Lewy pathology not only suggests a molecular and functional interaction between LRRK2 and α-synuclein but also provides support for the hypothesis that LRRK2 dysfunction may disrupt organelle trafficking and contribute to the pathogenesis of PD.

### 3.2. Roles of LRRK2 in Synucleinopathy

PD is now thought of as a complex multisystem disease with premotor and nonmotor symptoms, however, clinical-pathological diagnosis related to motor symptoms still remains the gold standard for the diagnosis of PD [120]. The current pathological criteria for PD require both DA neuronal loss in SNpc and LB pathology. Most LRRK2-assocaited PD patients are indistinguishable from idiopathic PD, both pathologically and clinically [121]. Pathological analyses revealed typical PD-type LB in most patients, but atypical neuropathology was also observed in some LRRK2 mutation carriers [122]. A subset of PD patients carrying a highly penetrant LRRK2 mutation, such as G2019S, R1441C/G, Y1699C, or I2020T, did not show LBs or α-synuclein pathology [120,123,124,125,126,127], despite the loss of DA neurons in SNpc [120]. However, it should be noted that the absence of LBs does not necessarily indicate that there is no α-synuclein-linked pathology. Native α-synuclein monomers can give rise to heterogeneous soluble oligomeric forms, which are not readily detectable in histological sections. Several lines of evidence suggest that LB themselves may be innocent bystanders in PD pathogenesis and that the neurotoxic species are in fact oligomers [128,129,130]. In this aspect, formation of LBs can represent a protective mechanism whereby insoluble fibrils function to sequester toxic oligomers [131]. It is a matter of debate whether LBs are neurotoxic or neuroprotective. Monitoring the dynamic process of α-synuclein aggregation rather than detecting the mere existence of LBs may be more informative to understand α-synuclein pathology. Levels of α-synuclein oligomers were significantly higher in the cerebrospinal fluid (CSF) of asymptomatic LRRK2 mutant carriers relative to healthy age-matched controls, suggesting a possibility that oligomeric α-synuclein is formed during the early stages of the disease prior to any major clinical manifestation and that LRRK2 might play a role in this pre-symptomatic stage by contributing to the formation of the toxic α-synuclein oligomers [132]. Supporting this notion, another study showed greater age-dependent accumulation of oligomeric α-synuclein in the striatum and cortex of LRRK2 R1441G KI mice at 15 and 18 months of age, compared to age-matched LRRK2 WT mice [133]. 

Attempts have been made to recapitulate Lewy pathology or synucleinopathy in rodent models and mimic the increased expression or phosphorylation of α-synuclein and/or the formation of insoluble α-synuclein aggregates or LB-like inclusions. In Tg mice overexpressing LRRK2 G2019S in catecholaminergic neurons under the control of TH promoter, DA and norepinephrine neurons degenerated in an age-dependent manner [52]. Moreover, elevated phosphorylation (pS129) of α-synuclein and high molecular weight species of α-synuclein were detected in the striatum and ventral midbrain of the LRRK2 G2019S Tg mice, whereas none of the pathogenic forms of α-synuclein were detected in nonTg or the LRRK2 G2019S/D1994A mice at 15 and 24 months of age [52]. LRRK2 G2019S KI mice showed age-dependent increases in phospho-α-synuclein (pS129) in SN and striatum [134]. However, most other Tg or KI mouse lines expressing pathogenic LRRK2 mutants under different promoter systems do not exhibit synucleinopathy, which may be in part explained by the insufficient amount of seeds required for α-synuclein aggregation. Tg mice expressing LRRK2 alone did not cause noticeable neurodegeneration, but the presence of excess LRRK2 accelerated the progression of neuropathological abnormalities developed in PD-related A53T α-synuclein Tg mice [135]. Co-expression of WT or LRRK2 G2019S with α-synuclein A53T caused synergistic toxicity to neurons that accelerated the progression of α-synuclein-mediated neuropathology. In another study, aged LRRK2 G2019S KI mice were more prone to develop α-synuclein toxicity than WT mice, and larger amounts of α-synuclein aggregates were present in aged LRRK2 G2019S KI mice [134,135]. Conversely, deletion of LRRK2 suppressed the aggregation and somatic accumulation of α-synuclein, and thereby delayed the progression of neuropathology developed in A53T Tg mice [135]. Preformed fibrils (PFFs) are artificially generated short fragments of fibrils that can trigger the conversion of endogenous α-synuclein into pathogenic fibril forms, and PFFs can function as seeds for the generation of insoluble fibrils or LB-like inclusions. In the α-synuclein PFF model, LRRK2 G2019S Tg mice showed accelerated α-synuclein aggregation, degeneration of DA neurons in SNpc, and neuroinflammation [136]. These lines of evidence suggest that LRRK2 may regulate α-synuclein-induced pathogenesis in PD. However, the link between LRRK2 and synuclein aggregation is far from clear and the literature is still controversial.

### 3.3. Roles of LRRK2 in α-Synuclein Propagation

The Braak model suggests that PD pathology spreads in a stereotypical fashion. According to the Braak model, PD pathology ascends caudo-rostrally from the lower brainstem and olfactory bulbs at the prodromal stage of disease, spreads through the midbrain and forebrain regions at disease diagnosis, and eventually propagates into the cerebral cortex at later stages [137]. Studies have suggested that α-synuclein exhibits prion-like properties and self-propagates via templating endogenous α-synuclein to form polymers [138]. The spreading of α-synuclein is considered to be an underlying molecular mechanism for the Braak hypothesis. Currently, cell-to-cell transmission of α-synuclein has been recapitulated in both cell culture and animal models [139,140,141,142]. The initial step of α-synuclein transmission may be the transformation of an innocuous, physiological form of α-synuclein into a toxic fibril, which may be caused by the misfolding of α-synuclein [128,129,130]. Infectious cells release α-synuclein oligomers or fibrils through undefined nonclassical secretory pathways, and α-synuclein then gains entry to nearby naïve cells [143], presumably by utilizing cell-to-cell communication mechanisms, such as passive diffusion, endocytosis, or transsynaptic transport [144,145]. Finally, the internalized pathogenic forms of α-synuclein act as seeds for de novo formation of α-synuclein fibrils, and thereby converts a naïve cell into an infectious cell. 

Multiple lines of studies have demonstrated that LRRK2 plays a role in the regulation of cell-to-cell transmission of α-synuclein. Kondo et al. [146] provided the first clues regarding a role of LRRK2 in α-synuclein transmission. Co-expression of LRRK2 G2019S and α-synuclein in human neuroblastoma SH-SY5Y cells increased α-synuclein aggregation and secretion when compared to cells expressing α-synuclein alone. Furthermore, more α-synuclein^+^ cells were detected after treatment with conditioned media collected from cells co-expressing LRRK2 G2019S and α-synuclein, when compared to cells treated with conditioned media collected from cells expressing α-synuclein alone. These results suggest that LRRK2 may be involved in the process of α-synuclein secretion and/or transmission. In an α-synuclein PFF-based transmission assay, where human α-synuclein PFFs trigger the aggregation of endogenous α-synuclein, α-synuclein aggregation was enhanced by LRRK2 G2019S compared to WT, but was decreased by the loss of LRRK2 in PD patient-derived neurons differentiated from induced pluripotent stem cells [136]. LRRK2 dependent α-synuclein transmission has also been demonstrated in multiple animal models. Bae et al. [147] generated *C. elegans* Tg models that expressed the N- and the C-terminal halves of the Venus fluorescent protein fused to α-synuclein in the pharyngeal muscles and the connected neurons, respectively, to assess intercellular α-synuclein transmission between pharyngeal muscles and neurons. Age-dependent increases in Venus fluorescence were detected in the WT background, but not in worms lacking *lrk1* (*Lrrk1* and *Lrrk2* ortholog in *C. elegans*). Bae et al. also injected AAV vectors encoding human α-synuclein into the vagus nerve of rats and tested neuron-to-neuron transmission of α-synuclein and long-distance protein spreading. The number of immunoreactive axons against α-synuclein were significantly decreased in the pons, the caudal and rostral midbrain, and the forebrain in rats lacking *Lrrk2* compared to WT. These studies imply that LRRK2 plays a role in cell-to-cell transmission and long distance spreading of α-synuclein, presumably through regulation of the release, uptake and/or lysosomal/proteosomal degradation of α-synuclein. The exact molecular mechanisms by which LRRK2 regulates α-synuclein transmission remain to be determined. 

**Figure 1:**
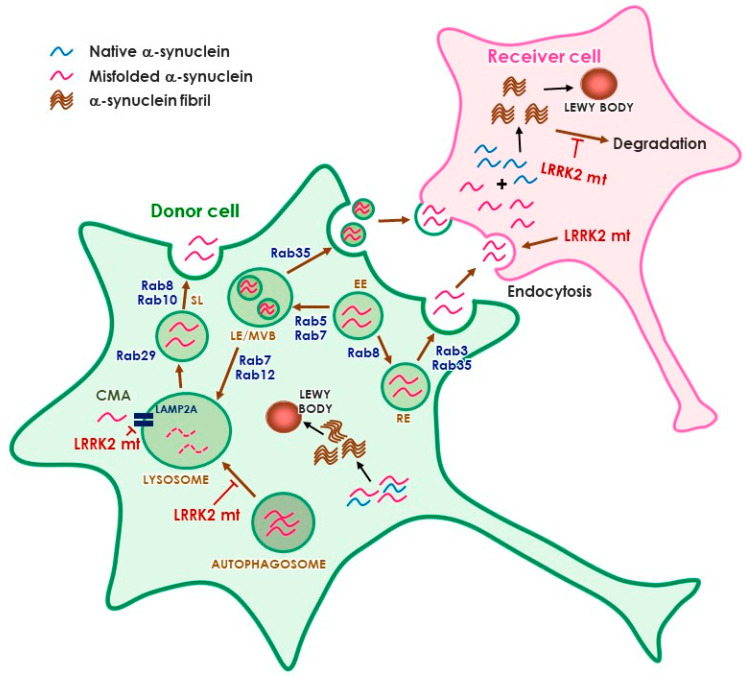
Potential role of LRRK2 in α-synuclein propagation. The cell may refer to neurons and/or glia. Abbreviations: EE, early endosome; LE, late endosome, MVB, multivesicular body, RE, recycling endosome, SL, stress lysosome, CMA, chaperone-mediated autophagy.

The identification of multiple Rab GTPases as physiological substrates of LRRK2 has raised an interesting hypothesis that links LRRK2 and α-synuclein transmission (Figure 1). In particular, Rab35, which has been suggested to mediate neurodegeneration induced by LRRK2 [80], has also been pointed out to mediate α-synuclein transmission stimulated by LRRK2 [147]. Rab35 could enhance the secretion and aggregation of A53T α-synuclein in SH-SY5Y cells [98]. How phosphorylation of Rab35 regulates α-synuclein propagation is unclear at this point. Considering the role of LRRK2 and Rab35 in endosomal recycling, it is plausible to suggest that activation of the LRRK2–Rab35 pathway would hijack the internalized α-synuclein aggregates from the endolysosomal degradation pathway, causing amplification of aggregates and continuous propagation [147]. Interestingly, several Rab GTPases that have been identified as modulators of α-synuclein toxicity, such as Rab1, 3, and 8 [148,149], are also LRRK2 substrates [80]. It is possible that more Rab GTPases are involved in LRRK2-mediated α-synuclein transmission. Rab8, 10, and 29 have been suggested as upstream and downstream molecules of LRRK2 in the regulation of endolysosomal pathway [97,150]. More studies are needed to define the link between LRRK2 and α-synuclein, but the LRRK2–Rab axis may have a critical role in α-synuclein transmission through the regulation of lysosomal degradation and secretory pathways.

## 4. Conclusions and Future Directions

Since the first descriptions of pathogenic mutations of LRRK2 in 2004 [151], remarkable advances have been made in our understanding of the function and dysfunction of LRRK2. Evidence so far suggests that LRRK2 plays multiple roles to control neuronal survival and death through cell-autonomous and non-cell-autonomous mechanisms. Apparently, the aberrant kinase activity of pathogenic LRRK2 mutants induces neurodegeneration by disturbing various intracellular processes, such as protein translation, endolysosomal pathway, autophagy, synaptic functions, and cytoskeleton dynamics, which may be mediated by the phosphorylation of several distinct putative substrates (Table 1). Although future studies are needed to identify the mechanistic link between LRRK2 and α-synuclein, pathogenic LRRK2 could exacerbate α-synuclein-mediated neurotoxicity, and LRRK2 has been suggested to interact with α-synuclein through intra- and inter-neuronal mechanisms. The available data suggest that targeting LRRK2 might be beneficial not only for patients with LRRK2 mutations but also for idiopathic PD patients. Therefore, LRRK2 has emerged as a promising target for potential disease-modifying therapies for PD, and clinical trials with small-molecule LRRK2 kinase inhibitors have already commenced. Until now, to understand LRRK2 pathology in PD, research interests have largely focused on the neurotoxic effects of pathogenic LRRK2 in the brain. Of note, accumulating lines of evidence suggest that LRRK2 plays a fundamental role in the regulation of inflammation in both the central and peripheral immune system [152,153]. Moreover, LRRK2 protein is also highly expressed in the kidney and the respiratory system. Therefore, increased knowledge of the role of LRRK2 in the immune system and in the periphery is needed and should be taken into consideration to develop effective and safe treatments for PD.

## Figures and Tables

**Table 1:** The list of LRRK2 kinase substrates and their potential functions.

Protein	Substrate Phosphorylation		Phospho-Site	Potential Role	Reference
	In Vitro	In Vivo			
ArfGAP1	↑ hLRRK2 WT, G2019S ↓ hLRRK2 KD	↓ Lrrk2 Knock-out (KO) mouse brain	S155, 246, 284 T189, 216, 292	GTPase activating protein (GAP) for LRRK2	[27]
β-tubulin	↑ hLRRK2 WT, G2019S ↓ hLRRK2 D1994A	ND	ND	A component of microtubule (MT) Neurite outgrowth	[67]
4E-BP-1	↑ dLRRK WT, Y1383C, I1915T ↑ hLRRK2 WT, I2020T ↓ dLRRK 3KD	↑ hLRRK2WT, I2020T in 293T cells ↓ hLRRK2 3KD in 293T cells	T37/46	Cap-dependent protein translation Survival under starvation, oxidative, and unfolded protein stress	[72]
Endophilin A	↑ hLRRK2 WT, G2019S ↓ hLRRK2 KD	↑ hLRRK2 WT, G2019S in CHO cells and *Drosophila* ↓ hLRRK2 KD in CHO cells and *Drosophila*	S75	Regulation of membrane curvature Synaptic vesicle endocytosis	[74]
FoxO1	↑ dLRRK ↑ hLRRK2 WT ↓ hLRRK2 3KD	↑ hLRRK2 WT, G2019S in 293T cells ↓ hLRRK2 3KD in 293T cells ↓ *dLRRK* null in *Drosophila*	S319	Transcriptional regulation of pro-apoptotic genes	[71]
Futsch	↑ hLRRK2 WT, G2019S ↓ hLRRK2 KD	ND	ND	Microtubule-association protein (MAP), regulation of MT dynamics Negative regulator of synaptic functions	[70]
MARK1	↑ hLRRK2 G2019S	↑ hLRRK2 WT, G2019S in HEK-293 cells ↓ hLRRK2 KD in HEK-293T cells	ND	Regulation of MT stability through phosphorylation of MAPs	[69]
Moesin/Ezrin/Radixin	↑ hLRRK2 WT, G2019S	↑ hLRRK2 WT, G2019S in HEK-293 cells	T558	Actin cytoskeleton rearrangement, neurite outgrowth, neuronal morphogenesis	[12,66]
NSF	↑ hLRRK2 WT, G2019S ↓ hLRRK2 KD	ND	T645	SNARE complex dissociation, synaptic vesicle endocytosis	[76]
P62/SQSTM1	↑ hLRRK2 WT, G2019S ↓ hLRRK2 KD	↑ hLRRK2 WT, G2019S, N1437, R1441G, Y1699C in HEK-293 cells ↓ hLRRK2 KD in HEK-293 cells	T138	Autophagy	[60]
Rab1a/b/c	↑ hLRRK2 WT, G2019S ↓ hLRRK2 KD	↑ hLRRK2 WT, G2019S in HEK-293 cells ↓ hLRRK2 KD in HEK-293 cells	T75	Endoplasmic reticulum (ER)-Golgi trafficking	[61,78,80]
Rab3a/b/c/d	↑ hLRRK2 WT, G2019S ↓ hLRRK2 KD	↑ hLRRK2 WT, R1441G, Y1699C, G2019S in HEK-293 cells	T94	Exocytosis, neurotransmitter release	[62,79,80]
Rab5a/b/c	↑ hLRRK2 WT	↑ hLRRK2 WT, R1441G, Y1699C, G2019S in HEK-293 cells	T6	Early and recycling endosomal trafficking	[61,65,78,79]
Rab8a/b	↑ hLRRK2 WT, G2019S ↓ hLRRK2 KD	↑ hLRRK2 WT, R1441C/G/H, Y1699C, I2020T, G2019S in HEK-293 cells	T72	Post-Golgi trafficking, ciliogenesis	[61,78,79,80]
Rab10	↑ hLRRK2 WT, G2019S ↓ hLRRK2 KD	↑ hLRRK2 WT, R1441C/G/H, Y1699C, I2020T, G2019S in HEK-293 cells	T73	Exocytosis, trans-Golgi/recycling endosome trafficking to plasma membrane	[62,78,79]
Rab12	↑ hLRRK2 WT, G2019S	↑ hLRRK2 WT, R1441G, Y1699C, G2019S in HEK-293 cells	S106	Recycling of endosomes and lysosomes, ciliogenesis	[63,64,78,79]
Rab29	↑ hLRRK2 WT	↑ hLRRK2 WT, R1441G, Y1699C, G2019S in HEK-293 cells	S72	Endolysosomal sorting/degradation	[61,78,79]
Rab35	↑ hLRRK2 WT, G2019S ↓ hLRRK2 KD	↑ hLRRK2 WT, R1441G, Y1699C, G2019S in HEK-293 cells	T72	Recycling endosomal trafficking, exosome secretion	[61,79,80]
Rab43	ND	↑ hLRRK2 WT, R1441G, Y1699C, G2019S in HEK-293 cells	T82	Anterograde ER-Golgi trafficking	[79]
RGS2	↑ hLRRK2 WT, G2019S, I2020T ↓ hLRRK2 KD	↑ hLRRK2 WT, G2019S in HEK293 cells cells ↓ hLRRK2 KD in ES derived human DA cells	ND	GAP for LRRK2	[29]
RPS15	↑ hLRRK2 WT, G2019S, I2020T ↓ hLRRK2 KD	↑ hLRRK2 WT, G2019S in ES derived human DA cells ↓ hLRRK2 KD in ES derived human DA cells	T136	Bulk protein translation	[73]
Snapin	↑ hLRRK2 WT, G2019S ↓ hLRRK2 KD	ND	T117	Synaptic vesicle trafficking	[75]
Synaptojanin-1	↑ hLRRK2 WT, G2019S ↓ hLRRK2 KD	ND	T1343,1348,1452,1503	Clathrin uncoating, down-regulation of actin polymerization, modulation of dynamin activity	[77]
Tau	↑ hLRRK2 WT, G2019S, I2020T	↑ hLRRK2 WT in SH-SY5Y cells ↓ hLRRK2 RNAi in SH-SY5Y cells	T181	Modulation of microtubule dynamics Neurite outgrowth	[68]

↑ increase; ↓ decrease; ND: Not defined.
